# IL-7 and CCR2b Co-Expression-Mediated Enhanced CAR-T Survival and Infiltration in Solid Tumors

**DOI:** 10.3389/fonc.2021.734593

**Published:** 2021-10-27

**Authors:** Guangchao Li, Qing Zhang, Zeping Han, Yangmin Zhu, Huijuan Shen, Zhi Liu, Zhao Zhou, Wen Ding, Siqi Han, Jinhua He, Zhao Yin, Jie Zhou, Ruiming Ou, Min Luo, Shuang Liu

**Affiliations:** ^1^ Department of Hematology, Guangdong Second Provincial General Hospital, Guangzhou, China; ^2^ Department of Research and Development, Guangzhou Bio-gene Technology Co., Ltd, Guangzhou, China; ^3^ Department of Laboratory, Central Hospital of Panyu District, Guangzhou, China; ^4^ Department of Medical Oncology, Jinling Hospital, Nanjing Clinical School of Southern Medical University, Nanjing, China; ^5^ Department of Hematology, People’s Hospital of Deyang City, Deyang, China

**Keywords:** CAR-T cells, IL-7, CCR2, immune cell therapy, neuroblastoma, melanoma

## Abstract

Chimeric antigen receptor T (CAR-T) cells are not effective in solid tumor treatment due to reduced invasion and expansion, and short survival time. This study aimed to explore whether interleukin (IL)-7 and CCR2b expression could improve GD2-CAR-T cell survival and infiltration in neuroblastoma and melanoma treatment. IL-7 and CCR2b were inserted into the classical second-generation CAR structure to construct 7×2b CAR. The 7×2b CAR-T cell phenotypes were evaluated by flow cytometry and the chemokine levels by ELISA. The 7×2b CAR-T cell migration and anti-tumor abilities were detected by Transwell assay and animal experiments *in vivo*. We report that compared with that of CAR-T cells, 7×2b CAR-T cell IL-7 secretion and CCR2b expression did not affect the T cell surface expression of CAR or CAR-T specificity and efficacy against tumor cells. The 7×2b CAR-T cells could induce IFN-γ secretion in GD2-positive tumor cells, killing them as well as conventional CAR-T cells. Moreover, IL-7 and CCR2b co-expression enhanced the 7×2b CAR-T cell survival and migration. Similar to conventional CAR-T, 7×2b CAR-T cells could also inhibit tumor growth and increase IFN-γ, Gzms-B, and IL-2 expression. Finally, unlike in mice injected with CAR-T cells, CD3 expression was the most abundant in the spleen and tumor tissues in mice injected with 7×2b CAR-T cells. Our study demonstrates that IL-7 and CCR2b co-expression in GD2-CAR-T cells exhibit stronger anti-tumor activity than classical second-generation CAR-T cells, shedding light on the potential novel GD2-positive neuroblastoma and melanoma treatment approach.

## Highlights

1. Successful 7×2b CAR-T cell construction.
2. IL-7 and CCR2b enhance CAR-T cell survival and migration.
3. 7×2b CAR-T cells have good anti-tumor activity.

## Introduction

Patients with cancer are known to be mainly treated by surgery, radiotherapy, and chemotherapy. Surgery is the best method for the removal of solid tumors while the metastatic or undetectable small lesions are mainly treated by radiotherapy and chemotherapy, destroying not only tumor tissues but also damaging healthy organs (1). Therefore, finding more effective methods to cure cancers is of utmost importance. Chimeric antigen receptor T (CAR-T) cells reportedly exhibit significant efficacy in cancer immunotherapy, especially in the treatment of blood cancers (2, 3). Recently, as a promising cancer strategy, FDA has approved 5 CAR-T cell therapies. However, CAR-T treatment in solid tumors suffers from certain bottlenecks, such as CAR-T survival in solid tumors and the effective CAR-T migration to solid tumors, that need to be resolved (4). The incorporation of new signal transduction domains into CAR-T cells, as well as exogenous cytokine and other genes co-expression with CAR could be a new strategy for improving CAR-T survival and their tumor-killing effect.

Human interleukin-7 (IL-7) is a pleiotrophic cytokine with a wide range of immune effects, which exhibits a direct or indirect role in the anti-tumor effect; it can also affect the growth, survival, and differentiation of B and T cells (5). At present, IL-7 application in immunotherapy has entered the clinical trial phase and achieved certain curative effects, including the cases of melanoma, lymphoma, and colon cancer (6–8). To date, clinical data have consistently demonstrated that IL-7 co-expression can prolong the survival time of CAR-T cells and improve their ability to expand and kill tumors (9). When exposed to IL-7, CAR-T cells reportedly exhibited improved persistence and antitumor activity *in vivo* (10). Moreover, IL-7 reportedly has a strong ability to amplify initial T cells and anti-tumor ability, while its side effects are scarce and could be tolerated by the patients (11), providing an opportunity for IL-7 use in tumor therapy.

In addition, metastatic T cell migration to the tumor is a multi-step process, requiring adhesion and integrin receptor expression for binding to the vascular wall, as well as chemokine receptors for detecting local chemokines and initiating migration (12). Monocyte chemoattractant protein-1 (MCP-1), as well as chemokine receptor type 2 (CCR2) and its ligand, are reportedly involved in inflammatory diseases, such as resistance to *Mycobacterium tuberculosis* during lung transplantation (13), lipopolysaccharide-related death (14), and delayed atopic dermatitis (15). CCR2 was also found to be one of the core receptors for the infection of human immunodeficiency virus type 1 (HIV-1) (16). CCR2b, one of the two isoforms encoded by CCR2, can migrate towards CCL2, a chemokine produced by various tumors. Therefore, CCR2b could be applied to enhance CAR-T migration in high-CCL2-expressing tumors, such as neuroblastoma and melanoma (17).

GD2, a disialoganglioside, is the most studied neuroblastoma-associated antigen that is highly and universally expressed on the neuroblastoma tissue (18). GD2-directed CAR-T cells have already been used in neuroblastoma treatment, although this approach also met obstacles preventing a significant effect (19).

In this study, we constructed and prepared GD2-targeted and IL-7 and CCR2b co-expressing CAR-T cells, also named 7×2b CAR-T cells. These cells display stronger anti-tumor activity, chemotactic ability, and subtype distribution compared to conventional CAR-T cells, providing a preclinical research basis for future clinical trials in solid tumor treatment.

## Materials and Methods

### Cell Lines

Human melanoma cell lines (SK-MEL-3, A375, C32, and Malme-3M cells) and human neuroblastoma cell lines (SK-N-SH, SK-N-AS, SK-N-MC, SH-SY-5Y, BE2-M17, and IMR-32 cells) were purchased from the American Type Culture Collection (ATCC). Dulbecco’s Modified Eagle Medium (DMEM; Gibco, USA) supplemented with 1 % penicillin/streptomycin and 10 % fetal bovine serum (FBS; Invitrogen, Carlsbad, CA, USA) was used to culture cells at 37°C with 5% CO_2_.

### Bioinformatics Analysis

The GSE96631 dataset, downloaded from https://www.ncbi.nlm.nih.gov/geo/, was used to compare the expression of CCL2 in neuroblastoma, healthy adrenal gland, and embryonic neural cristae cells.

### CAR Construction

The anti-GD2 scFv was derived from the murine monoclonal antibody 14.G2a (WO 2013/040371). GD2 CAR containing 14.G2a scFv, CD3ζ domain along with 4-1BB co-stimulatory domain was generated as described previously (20). The sequence was codon-optimized for expression in human cells and synthesized by Guangzhou IGE Biotechnology, then cloned into a pLVX lentiviral expression vector (the EcoRI and BamHI sites). For the 7×2b CAR, the full-length IL-7 and CCR2b sequences were inserted at the C-terminal of the GD2 CAR and separated by the self-cleaving 2A peptide. The final CAR construction sequence was verified and used for downstream applications.

### Lentiviral Particles and CAR-T Cell Transduction

CAR-encoding lentiviral particles were produced by transient transfection of Lenti-X 293T cells (TaKaRa) as described previously (21). Briefly, the CAR-expressing plasmid, along with 3 packaging plasmids (pLP1, pLP2, and pLP/VSVG), was transfected into 293T cells by polyetherimide (PEI, Sigma-Aldrich). Supernatants were collected 48 h post-transfection and used for gene transduction after ultracentrifugation. T cells were activated by anti-CD3/CD28 beads (Dynabeads, Life Technologies), then infected with lentiviral particles in the presence of RetroNectin (Takara Bio, Shiga, Japan). GT-T551 H3 culture medium (Takara) with 4% Human AB serum and 300 U/mL IL-2 were used to culture CAR-T cells. Peripheral blood mononuclear cells (PBMCs) and healthy donor T cells were obtained from Guangzhou Leide Bioscience Co., Ltd with ethical approval.

### Flow Cytometry

For the detection of the GD2 expression, tumor cells were stained with an anti-Ganglioside GD2 antibody (14.G2a, Abcam; ab68456). For the detection of the expression of GD2 CAR and chemokine receptors, a Goat F(ab’)2 Anti-Mouse IgG (Fab’)2 (FITC) (Abcam; ab98658) was used to stain T cells, FITC-mouse anti-human CCR2 (Biolegend; 357216). For the detection of the Tscm cells expressing CD45RA, CD62L, CCR7, a PE-Cy7-conjugated anti-CD62L antibody (BioLegend, 104417), a PerCP-Cy5.5-conjugated anti-CCR7 antibody (BioLegend, 120115) and a Brilliant Violet 421-conjugated anti-CCR7 antibody (SONY, 1200595) were used to stain the T cells. We used a CytoFLEX Flow Cytometer (Beckman Coulter, CA, USA) to select the Data and the CytExpert Software (Beckman) or FlowJo version 7.6.1 (Tree Star, Inc.) to analyze it.

### Chemokine Enzyme-Linked Immunosorbent Assay (ELISA)

To measure the human IL-7 production by the CAR-T cells, 1×10^6^ T cell supernatants were collected. We measured the chemokine levels using a human IL-7 ELISA kit (CHE0143, Beijing 4A Biotech) according to manufacturer instructions. IFN-γ levels were determined by analyzing supernatants from triplicate wells following 16 h of 1×10^4^ target and 1×10^5^ effector T cell co-culture using a human IFN-γ ELISA kit (CHE0017). To measure human CCL2 production, 1×10^6^ tumor cells were plated into 6-well plates; the supernatant was collected after 24 h of incubation and processed using a human MCP-1 ELISA kit (CHE0103). The mouse serum (IFN-γ, granzyme B, IL-2, IL-7) was quantified by a cytometric bead array (CBA; Becton Dickinson Biosciences) according to manufacturer instructions.

### Cellular Cytotoxicity

Luciferase-based reporter assays were performed to determine GD2 CAR-T cell activity against tumor cell targets using the Bright-Glo Luciferase Assay System (Promega; E2620). We seeded 1×10^4^ target cells (SK-N-AS, IMR-32, A375) expressing the luciferase gene, just as well as effector T cells, into 96-well black assay plates (Shanghai Jinan; J09602). After co-culture for 4 h, 100 μL substrate-supplemented buffer was added in the wells and measured using FLUOstar Omega (BMG LABTECH Inc, NC, USA).

### *In Vitro* Transwell Assay

We starved 7×2b CAR-T, GD2 CAR-T, and Mock T (3×10^4^) cells overnight, and then seeded them in the upper chamber of a Transwell (5 μm pore size; Costar Transwell, Corning, NY) while the lower chambers contained 600 μL of serum-free culture supernatants from 293T or SK-N-AS cells. We used serum-free medium with 10 ng/mL of recombinant CCL2 (R&D Systems, Minnesota, USA) as a positive control. The migrated were collected from cells in the lower chambers after 12 h of incubation; CountBright Absolute Counting Beads (Invitrogen) were added into the wells, and the cells were counted with a Beckman Coulter CytoFlex flow cytometer (Beckman Coulter).

### Mice and *In Vivo* Tumor Models

Animal studies were carried out under protocols approved by the Animal Care and Use Committee of Guangzhou Institutes of Biomedicine and Health (GIBH). B-NDG (NOD-Prkdcscid IL2rgtm1/Bcgen) immune-deficient mice were purchased from Biocytogen (Jiangsu, China). IMR-32 cells (5×10^6^) expressing CCL2 (IMR-32-CCL2) were injected subcutaneously (s.c.) in the back of the mice. Mock T, classical GD2 CAR-T, and 7×2b CAR-T cells (3×10^6^) were administered intravenously 10 d after tumor inoculation. We performed the intraperitoneal injection of 3 mg D-luciferin (Perkin-Elmer) every 7 d for the *in vivo* imaging using the IVIS system (Caliper Life Science), and the blood samples were obtained from the tail vein. The spleen and tumor sampling of satellite mice were performed on 7 d post-treatment, and the expansion and infiltration of CAR-T were evaluated using anti-human CD3 *in vivo* by immunohistochemical assays.

### Statistical Analysis

The statistical analysis was conducted using GraphPad Prism 8 software. The results were expressed as the mean ± standard deviation (mean ± SD), and all experiments were independently performed in triplicates. Two- or one-factor analysis of variance was performed for the comparison between the groups. We used the independent samples t-test for the comparison between the two groups. P-values of *p* < 0.05 were considered statistically significant.

## Results

### IL-7 and CCR2b Co-Expression in Anti-GD2 CAR-T Cells

**Figure 1:**
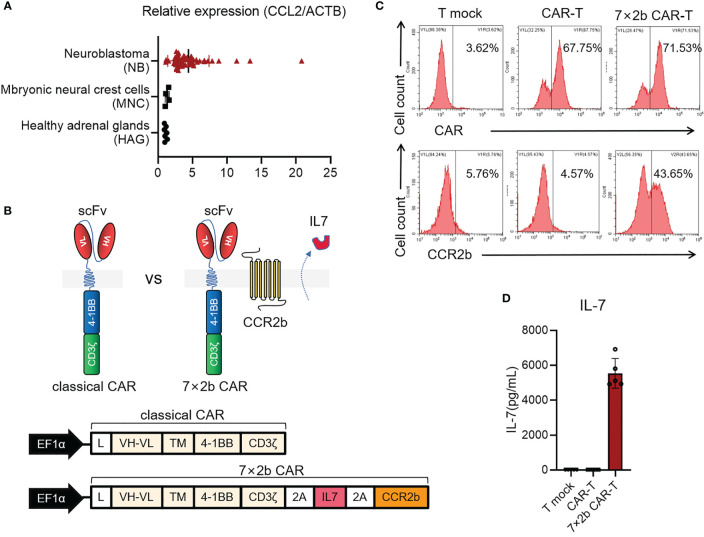
IL-7 and CCR2 co-expression in anti-GD2 CAR-T cells. **(A)** The mRNA expression of CCL2 was analyzed in GSE96631 from the GEO database. **(B)** Structure diagram of 7×2b CAR. **(C)** The expression of CAR and CCR2b in CAR-T cells. **(D)** The T-mock and CAR-T cells were cultured in a H3 medium without IL-7. IL-7 levels in the supernatant on day 5 were detected by ELISA.

First, we observed a high level of CCL2 expression in neuroblastoma compared with that in the healthy adrenal gland and embryonic neural cristae cells (**Figure 1A**, *p* < 0.05) by analyzing the GSE96631 dataset. To recruit more CAR-T cells to the tumor sites with high CCL2 expression and maintain their long-term survival, the GD2 antigen-targeting CAR structure was modified. As shown in **Figure 1B**, the full-length IL-7 and CCR2b sequences (360aa) were inserted into the classical second-generation CAR structure, separated by the 2A self-cleaving peptide. Our results showed that 7×2b CAR-T cells expressed not only CAR but also CCR2b (**Figure 1C**) and effectively secreted IL-7 (**Figure 1D**, *p* < 0.05).

### Anti-Tumor Activity of 7×2b CAR-T Cells *In Vitro*

**Figure 2:**
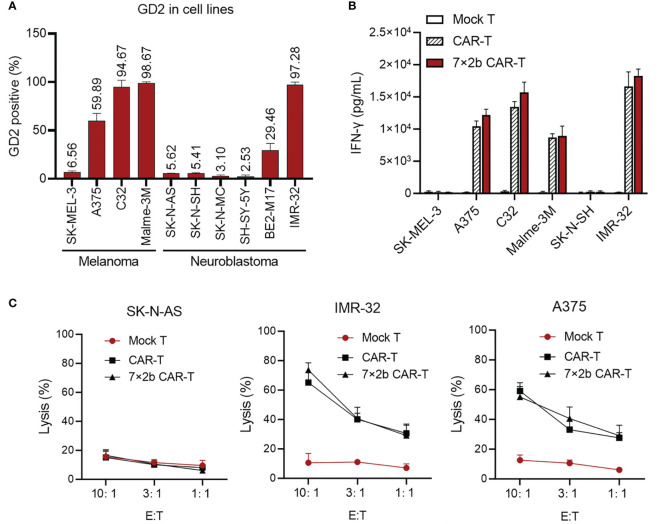
The effect of 7×2b CAR-T cells on tumor cells. **(A)** The expression of GD2 in melanoma and neuroblastoma cell lines. **(B)** After co-incubation for 12 h at a ratio of 10:1, the supernatant was removed to detect the IFN-γ secretion level by ELISA. **(C)** Luciferase-based reporter assays were used to detect the lysis (%) of 7×2b CAR-T cells against three melanoma and neuroblastoma cells with different E:T ratios.

To investigate the 7×2b CAR-T cell anti-tumor activity, we detected the expression of GD2 in melanoma and neuroblastoma cells. As shown in **Figure 2A**, we observed a high expression of the GD2 antigen in the melanoma (A375, C32, and Malme-3M) and neuroblastoma (BE2-M17 and IMR-32) cells. By co-culturing 7×2b CAR-T cells, T cells, and CAR-T cells with GD2-positive tumor cells (A375, C32, Malme-3M, and IMR-32), we found that both classical and 7×2b CAR-T cells secreted a significant amount of IFN-γ, while the GD2-negative tumor cells (SK-MEL-3 and SK-N-SH) could hardly induce the release of IFN-γ (**Figure 2B**). In addition, both classical CAR-T and 7×2b CAR-T cells killed GD2-positive IMR-32 and A375 cells, but did not kill GD2-negative SK-N-AS cells, exhibiting high specificity with equivalent efficiency *in vitro* (**Figure 2C**). Taken together, these results suggest that 7×2b CAR-T cells could be specifically activated by GD2-expressing tumor cells, exhibiting excellent tumoricidal effects.

### The IL-7 and CCR2b Co-Expression Enhanced 7×2b CAR-T Cell Survival and Migration

**Figure 3:**
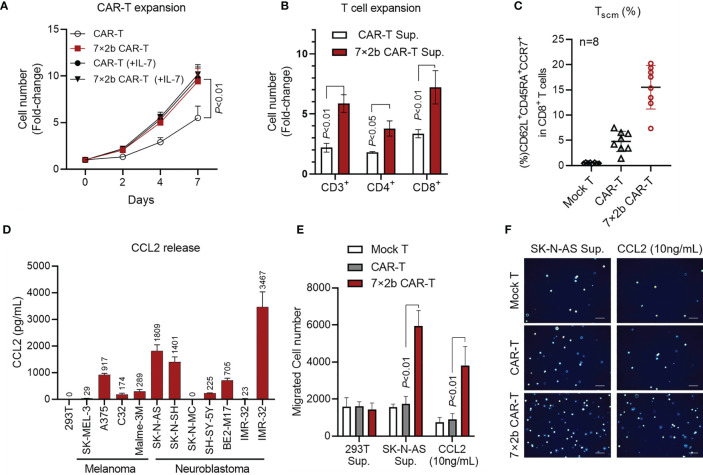
The IL-7 and CCR2b co-expression enhanced 7×2b CAR-T cell survival and migration. **(A)** The proliferation ability of 7×2b CAR-T cells. We cultured one million conventional CAR-T and 7×2b cells (all with IL-2), and the cell number was calculated in the presence or absence of IL-7. **(B)** The effect of 7×2b CAR-T cell supernatant on T cell amplification. Supernatants of CAR-T were collected and mixed with H3 medium supplemented with 2% of autologous serum and 300 U/mL of IL-2 at a 1:1 ratio, which was then used to culture 1×106 CD3+ T cells for 7 days to detect the amplification ratio of CD3, CD4, and CD8 subsets. **(C)** The CAR-T cells were prepared independently 8 times, and the TSCM subset proportions were analyzed. **(D)** The CCL2 level in the supernatant was detected by ELISA after 48 h of culture of 5×106 tumor cells. **(E)** The migration ability of CAR-T cells induced by HEK293T cells, SK-NS-AS cells, and CCL2 (100 ng/mL). **(F)** Representation of the lower Transwell compartment, showing T cells in each lower compartment. N = 3, p < 0.05, p < 0.01.

To further verify 7×2b CAR-T cell function, we examined the CAR-T cell proliferation ability. As shown in **Figure 3A**, from day 2, the proliferation ability of the antigen-activated 7×2b CAR-T cells was significantly higher than that of classical CAR-T cells (*p* < 0.01). Moreover, we found that IL-7 was could significantly improve the CAR-T cell, but not the 7×2b CAR-T cell proliferation ability, indicating that the IL-7 secreted by the 7×2b CAR-T cells was sufficient to meet cell proliferation requirements. Furthermore, we evaluated the role of the CAR-T cell supernatant in T cell amplification and found that the supernatant of both classical and 7×2b CAR-T cells could amplify CD3^+^, CD4^+^, and CD8^+^ T cell subsets, but the number of the amplified CD8^+^, CD3^+^, and CD4^+^ T cells in the 7×2b CAR-T cell sample was higher than that in the classical CAR-T cell sample (**Figure 3B**, *p* < 0.05, *p* < 0.01). Moreover, compared with the mock T and conventional CAR-T cells, the T memory stem cell proportion (T_SCM_) in CD8^+^ T cells (CD62L^+^CD45RA^+^CCR7^+^) in the 7×2b CAR-T cells was significantly higher (**Figure 3C**, *p* < 0.01), indicating that 7×2b CAR-T cells exhibit a potential persistence. In addition, as shown in **Figure 3D**, several melanoma and neuroblastoma cells were found to be able to secrete large amounts of CCL2, including SK-NS-AS and IMR-32-CCL2 cells (IMR-32 cells stably expressing CLL2). Furthermore, compared with the mock and CAR-T cells, the SK-NS-AS cells induced the highest migration rate of the 7×2b CAR-T cells (**Figures 3E, F**, *p* < 0.01), suggesting the chemotactic effect of 7×2b CAR-T cells to the supernatant containing CCL2.

### Anti-Tumor Activity of 7×2b CAR-T Cells *In Vivo*

**Figure 4:**
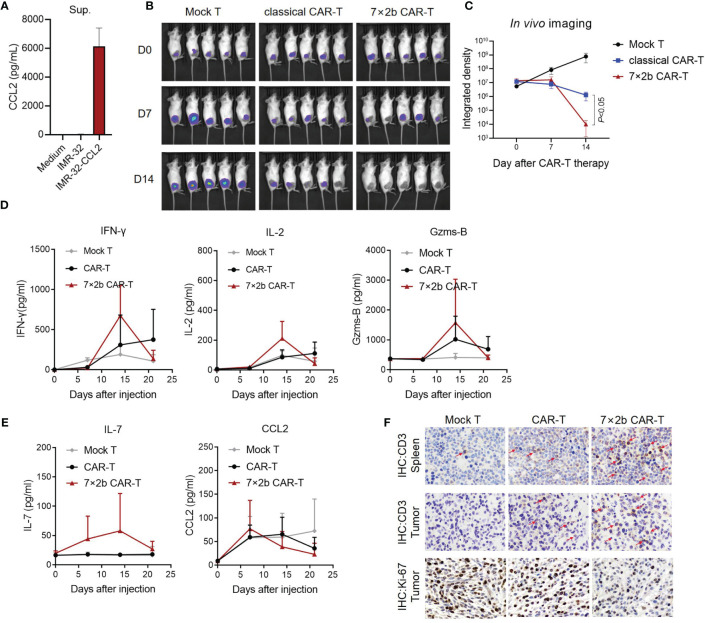
Anti-tumor activity of the 7×2b CAR-T cells *in vivo*. **(A)** The level of CCL2 secretion in IMR-32-CCL2 cells was detected by ELISA. **(B)** The antitumor activity of CAR-T cells was evaluated by *in vivo* imaging in mice bearing IMR-32-CCL2 xenografts. **(C)** Fluorescence evaluation of the *in vivo* imaging of the mice in **(B)**. **(D)** The levels of IFN-γ, IL-2, and GZMS-B in the sera of the mice were detected by ELISA. **(E)** IL-7 and CCL2 were also measured. **(F)** The human CD3 and Ki-67 positive cells in the spleen and tumor of the mice were detected by immunohistochemistry after CAR-T infusion. N = 5.

Finally, we evaluated how the 7×2b CAR-T cells affect the tumor *in vivo*. We used IMR-32 to construct a stable cell line (IMR-32-CCL2) to express CCL2 and luciferase, and CCL2 secretion was detected by ELISA (**Figure 4A**). After subcutaneous tumor formation with IMR-32-CCL2, mice were treated with CAR-T injection through the caudal vein. We found that, unlike the mock T cells and classical CAR-T cells, 7×2b CAR-T cells could significantly inhibit the tumor growth (**Figures 4B, C**). Moreover, IFN-γ, IL-2, and GZMS-B increased significantly on day 14 after the CAR-T treatment, consistently with the timing of the CAR-T response *in vivo* (**Figure 4D**). IL-7 was found to only present in the venous blood of mice with an injection of 7×2b CAR-T cells, indicating that IL-7 was secreted by the 7×2b CAR-T cells (**Figure 4E**). CCL2 was secreted primarily by the IMR-32-CCL2 xenografts, and the levels were essentially the same in all three mouse groups on day 7 with subsequent changes appearing to correlate with the degree of tumor subduction (**Figure 4E**). In addition, *in vivo* CAR-T cell infiltration and expansion were further assessed by detecting the proportion of CD3 in the spleen and tumors of the mice. More CD3 positive cells were found in mice treated with 7×2b CAR-T cells compared with that in those treated with classical CAR-T cells and mock T cells (**Figure 4F**), suggesting that 7×2b CAR-T cells had a strong amplification ability and were more adapted to migrate to tumor sites. We also found that the Ki67 expression were most decreased in 7×2b CAR-T cells than normal CAR-T, it means that 7×2b CAR-T cells have a better antitumor effect than normal CAR-T (**Figure 4F**).

## Discussion

Although significant breakthroughs have been made in the treatment of hematological malignancies, the CAR-T cell therapy in solid tumor treatment still faces challenges, mainly due to the antigenic heterogeneity of the cancer cells (22), complexity of the cellular components constituting the solid tumors, insufficient infiltration ability, migration of CAR-T cells intotumor tissues, and immunosuppressive conditions in the tumor microenvironment (23).

To solve these problems, we constructed a second-generation CAR structure targeting GD2 (7×2b) and co-expressing IL-7 and CCR2b in the CAR-T cells. It has an obvious superior function in proliferation, migration, subtype distribution, and tumor-killing compared with conventional GD2 CAR-T cells, which is expected to provide a new therapeutic strategy for GD2-positive neuroblastoma and melanoma.

IL-7 secretion and CCR2b expression did not affect the expression of CAR on the T cell surface and the specificity and effectiveness of CAR-T against tumor cells. IL-7 is known to have a positive effect on memory phenotype maintenance and CAR-T proliferation during *in vitro* CAR-T culture (9). However, addition of IL-7 during *in vitro* culture increases the cost of preparation and is not suitable consistently for clinical applications of CAR-T. Here, we satisfy the *in vivo* and *in vitro* need for IL-7 by co-expressing it with CAR, the coexpression of IL-7 with CAR-T cells is safety. Furthermore, without exogenous IL-7 addition, 7×2b CAR-T cells showed better proliferation ability, which would be conducive to their survival *in vivo* and improve their clinical activity. In addition, the CAR-T phenotype reportedly exhibits a clinical curative effect of a key factor (24); T memory stem cells are less differentiated and have a greater ability to differentiate into subsets of all memory and effector T cells and self-renew compared with the central memory T cells, which are thought to have a stronger anti-tumor activity and stamina (25, 26). Studies have shown that IL-7 tends to induce CAR-T cell differentiation to generate naive, T_SCM_, and other cells with lower differentiation degrees (27). In our study, cell subtype analysis showed that the T_SCM_ ratio in 7×2b CAR-T cells was indeed higher than that of conventional CAR-T, which is conducive to the formation of long-term tumor-specific memory function *in vivo*, and thus more effective in preventing tumor recurrence.

Studies have shown that improving CAR-T cell ability to migrate to tumor lesions increases their anti-tumor immune response (28). By binding tumor-produced chemokines to appropriate CCR on injected activated T cells, T cell transport can be enhanced. A mismatch between chemokines secreted from tumors and appropriate CCRs expression on T cells will result in suboptimal trafficking (29). Considering that adoptively transferred T cells are genetically modified by inserting optimized T cell receptors or CARs, it is reasonable to hypothesize that additional modifications that alter chemokine receptor expression may be advantageous. In order to better match the chemokine secreted by tumor cells, some studies have introduced chemokine receptors such as CCR2b into CAR-T cells through genetic modification and promoted the transport by the large amount of CCL2 secreted by tumor cells (30). The catalytic factor CCR2b is a chemokine of CCL2. In this study, we demonstrated that CCL2 secreted by tumor cells promotes the migration of 7×2b CAR-T cells, which contributes to 7×2b CAR-T cell recruitment to the tumor site *in vivo* to fight the tumor. In addition, *in vivo* studies of a subcutaneous xenograft model of neuroblastoma in nude mice showed that 7×2b CAR-T cells effectively cleared tumors in mice, and also secreted a large number of human IFN-γ, IL-2, and GZMS-B, suggesting 7×2b CAR-T cells have a powerful anti-tumor effect.

In summary, our study confirms the potent 7×2b CAR-T cell antitumor effects against neuroblastoma and melanoma. However, more detailed mechanisms need to be further investigated to indicate 7×2b CAR-T cell anti-tumor activities in patient-derived xenograft (PDX) models or mice engrafted with human PBMCs. This research provided a preclinical basis for subsequent clinical trials.

## Data Availability Statement

The original contributions presented in the study are included in the article/supplementary material. Further inquiries can be directed to the corresponding authors.

## Ethics Statement

Animal studies were carried out under protocols approved by the Animal Care and Use Committee of Guangzhou Institutes of Biomedicine and Health (GIBH).

## Author Contributions

SL, RO, ML, GL, QZ, ZL, ZH, and JH conceived and designed the project. HS, YZ, ZZ, WD, SH, and JZ collected the data, performed the interpretation of data and statistical analysis. GL, QZ, and ZL wrote the manuscript. SL, ZY, RO, and ML revised the paper. All authors contributed to the article and approved the submitted version.

## Funding

This study was supported by the Guangzhou Science and Technology Plan Project (No. 202002030404), the Foundation of Guangdong Second Provincial General Hospital (No. 2017-001, 3DB2020014, YQ2020-002), Doctoral Workstation Foundation of Guangdong Second Provincial General Hospital (No. 2019BSGZ008, 2020BSGZ048, 2021BSGZ017), Guangdong Medical Scientific Research (No. B2020092) and the Guangzhou Bio-gene Co., Ltd and Pearl River S&T Nova Program of Guangzhou from Guangzhou Municipal Science and Technology Bureau (No. 201906010056), The Medical and Health Science and Technology Project of Panyu District, Guangzhou (No.2018-Z04-59); Natural Science Foundation of Guangdong Provience (No. 2021A1515012329) and Foundation of Deyang People’s Hospital (No. FHT202004). The funder was not involved in the study design, collection, analysis, interpretation of data, the writing of this article or the decision to submit it for publication.

## Conflict of Interest

Authors GL, ZZ, WD and ML were employed by Guangzhou Bio-gene Technology Co., Ltd.

The remaining authors declare that the research was conducted in the absence of any commercial or financial relationships that could be construed as a potential conflict of interest.

## Publisher’s Note

All claims expressed in this article are solely those of the authors and do not necessarily represent those of their affiliated organizations, or those of the publisher, the editors and the reviewers. Any product that may be evaluated in this article, or claim that may be made by its manufacturer, is not guaranteed or endorsed by the publisher.
